# Contribution to the cytogenetics of Kuwaniini scale insects (Homoptera, Coccinea, Margarodidae s.l.)

**DOI:** 10.3897/CompCytogen.v11i4.20168

**Published:** 2017-09-14

**Authors:** Ilya Gavrilov-Zimin

**Affiliations:** 1 Zoological Institute, Russian Academy of Sciences, St. Petersburg, Russia

**Keywords:** *Jansenus burgeri*, scale insects, morphology, life cycle, karyotype, sex chromosome system

## Abstract

*Jansenus burgeri* Foldi, 1997 (Margarodidae s.l., Xylococcinae, Kuwaniini) was studied cytogenetically for the first time. It was shown that the species reproduces bisexually, displays XX/X(0) sex chromosome system and 2n=6/5 (female/male) including two pairs of long autosomes and a pair of shorter X-chromosomes in female. The chromosome complement, adult female morphology and the life cycle of *J. burgeri* are illustrated. The cytogenetic data are in fact the first ones for Kuwaniini scale insects, because *Kuwania oligostigma* De Lotto, 1959 briefly cytogenetically studied by Hughes-Schrader (1963), in my opinion, may be excluded from the genus *Kuwania* Cockerell, 1903 and the tribe Kuwaniini, since this species shows aberrant morphological characters, specifically the total absence of abdominal spiracles and the presence of tubular ducts.

The tribe Kuwaniini MacGillivray, 1921 (Margarodidae s.l., Xylococcinae), which currently comprises four nominal genera (Wu and Nan 2012), has not been explored cytogenetically up to the present excluding the brief note (without photographs) of Hughes-Schrader (1963) on chromosomal number (2n=16) in *Kuwania oligostigma* De Lotto, 1959, whose taxonomic position is questionable (see below). Two other genera of the tribe, *Neogreenia* MacGillivray, 1921 and monotypic *Neosteingelia* Morrison, 1927, have never been studied in terms of cytogenetics and reproductive biology. In 1997 Dr. Imre Foldi described from Thailand a peculiar new species and new monotypic genus, *Jansenus burgeri* Foldi, 1997, placed by him in the subfamily Xylococcinae without tribal attribution. Wu and Nan (2012) considered this genus as closely related to *Kuwania*, *Neogreenia* and *Neosteingelia*, and that conforms to my own view on the taxonomy of this group.

The exact type locality (in Thailand) for *Jansenus burgeri* was unknown and there have been no reports on new findings of this species since its original description. In June 2017 I was able to collect fresh material on this species during my expedition in Northern Laos (bank of Mekong, Pak Beng village, on stem under the bark of undetermined tree, adult females and larvae, guarded by ants, 14.VI.2017, I. Gavrilov-Zimin, collection number K 1385, preserved at Zoological Institute, Russian Academy of Sciences). The material was suitable both for morphological investigation (Fig. 1) and study of karyotype and chromosome system in this species. The chromosome counts were performed in young embryos of both sexes, squashed in a drop of lactoaceticorcein. *J. burgeri* was found to reproduce bisexually, have XX/X(0) chromosome system and 2n=6/5 (Fig. 2); diploid karyotype includes two pairs of long autosomes and a pair of shorter X-chromosomes in females while one X-chromosome in males. The mature female lays eggs before cleavage divisions in the white cottony wax sac suggesting thus the normal oviparity. As other Xylococcinae and some other Margarodidae s.l., *J. burgeri* has apodal cyst-like stages in its life cycle (Fig. 3).

Only a few species of Xylococcinae have been studied cytogenetically till now and all available data were published without photographs of the chromosomes. Thus, *Kuwania oligostigma* De Lotto, 1959 (tribe Kuwaniini) shows 2n=16 in adult female and bisexual reproduction (Hughes-Schrader 1963); the chromosome number in males and in embryos of both sexes were not studied. Meanwhile, the morphology of *K. oligostigma* is significantly different from that of other species of the genus and other genera of the tribe. According to the original morphological description and provided figure, *Kuwania oligostigma* has totally lost the abdominal spiracles (a unique situation for Xylococcinae!) and has tubular ducts in contrast to all other Kuwaninii. In my opinion, the species may be excluded from the tribe, but its correct taxonomic placement remains obscure for me.

**Figure 1.**
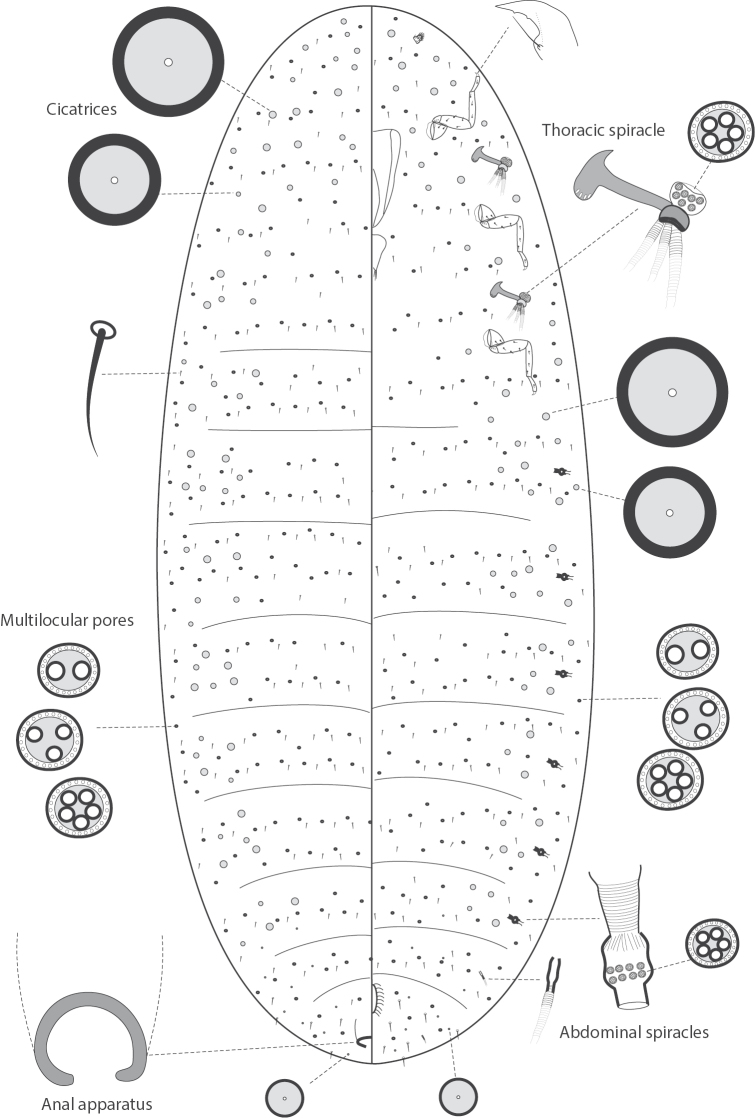
Morphology of adult female of *Jansenus burgeri* Foldi, 1997, Laos (Pak Beng).

**Figure 2.**
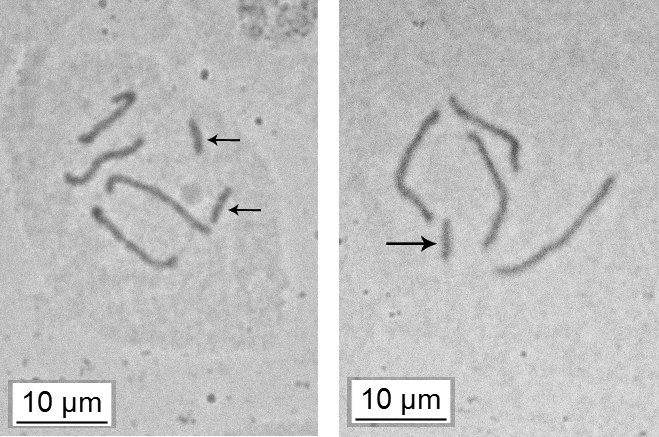
Embryonic cells of *Jansenus burgeri* Foldi, 1997 in female (2n=6) and male (2n=5) embryos; sex chromosomes are arrowed.

**Figure 3.**
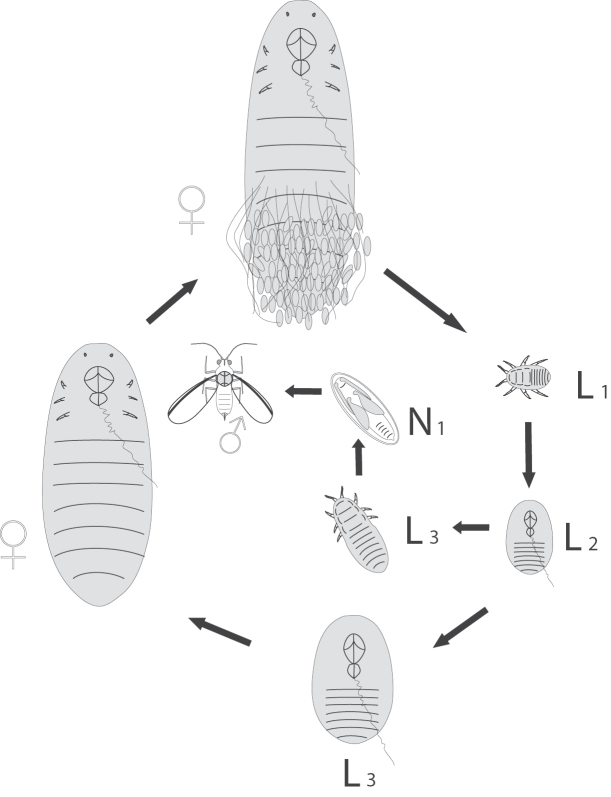
The life cycle of *Jansenus burgeri* Foldi, 1997; L_1_–L_3_ – first, second and third larval stages; N_1_ – nymphal (preimaginal) stage with protoptera (wing buds) of male.

*Matsucoccus gallicolus* Morrison, 1939 (Xylococcinae, ) was studied by Hughes-Schrader (1948) who reported for this species XX-X(0) chromosome system with multiple X chromosomes, i.e., 2n=28A+12X in females and 2n=28A+6X in males.

Nur (1980) studied gravid females of *Steingelia gorodetskia* Nasonov, 1908 (Xylococcinae, Steingeliini) and found that the species had the bisexual reproduction, XX-X(0) chromosome system and 2n=10 in females.

Thus, amongst the four Xylococcinae species studied so far, *Jansenus burgeri* shows the lowest chromosome number, 2n=6. Of the other scale insects, the same number is known only in few species of the subfamily Monophlebinae (Orthezioidea, Margarodidae s.l.) and in some species of neococcids (superfamily Coccoidea) – see for review Gavrilov 2007.
